# Research on mechanical automatic food packaging defect detection model based on improved YOLOv5 algorithm

**DOI:** 10.1371/journal.pone.0321971

**Published:** 2025-04-24

**Authors:** Guanyong Liu, Shuai Zhang, Lixin Wang, Xiaoran Li, Gongchen Li

**Affiliations:** Internship and Training Management Office, Binzhou Polytechnic, Binzhou, Shandong, China; G H Raisoni College of Engineering and Management, Pune, INDIA

## Abstract

With the rapid development of industrial automation, traditional manual detection methods are inefficient and error-prone, which cannot meet the needs of modern production for high efficiency and high precision. Therefore, it is particularly important to develop a mechanical automatic inspection system that can automatically identify food packaging defects. In this study, aiming at the limitations of existing technologies in identifying small targets and subtle defects, an enhanced YOLOv5-based model for detecting food packaging flaws is introduced. Firstly, we integrated a Convolutional Attention module (CBAM) to enhance the model’s attention on crucial image features. This mechanism prioritizes significant features by weighting the feature map in channel and spatial dimensions, which improves accuracy in detecting minor defects and small objects. Secondly, feature fusion across scales is achieved with pyramid and aggregation networks, so that the model can capture defects of different sizes at the same time, which enhances the recognition ability of diverse defects in food packaging. In addition, this study also optimizes the backbone network structure of YOLOv5. By integrating the streamlined YOLOv5s model and adding an Adaptive Spatial Feature Fusion module (ASFF), the model’s ability to blend features from different scales was enhanced. In this study, 7400 images with 512×512 resolutions were applied to develop the proposed model. The experimental results show that the improved model outperforms the original YOLOv5 model in terms of Accuracy (Ac), Recall (Re), and F1 score, with values of 0.96, 0.94, and 0.94, respectively, effectively improving the automation and accuracy of food packaging defect detection when compared with YOLOv5+ASFF (Ac=0.94, Re=0.95, and F1=0.94), original YOLOv5 (Ac=0.82, Re=0.85, and F1=0.88), and YOLOv5+CBAM (Ac=0.88, Re=0.9, and F1=0.89). Additionally, the present performance of an improved YOLOv5 model (CBAM+Fusion Pyramid Network (FPN)+Path Aggregation network (PANet)+ASFF) was significantly comparable to the related research works.

## 1. Introduction

With the rapid development of the global food industry, the quality and safety issues of food packaging are increasingly receiving attention. Food packaging defect detection refers to the systematic identification and classification of various defects that occur in packaging materials or packaging processes in the field of food processing and packaging, such as holes, cracks, poor sealing, labeling errors, or irregularities. These not only affect the appearance quality of products, but may also become potential pathways for food contamination. Therefore, achieving automated and efficient food packaging defect detection is of great significance for ensuring food safety and improving production efficiency.

With the advancement of technology, computer vision technology has provided an automated alternative solution for food packaging defect detection. Computer vision is a science that enables computers to extract information from images or multidimensional data. Its application in food packaging defect detection can greatly improve detection speed and accuracy. Image-based automatic detection methods can identify and classify defects by analyzing images without the need for manual intervention, thereby reducing human errors [1].

In the field of computer vision, deep learning technology, and the YOLO algorithm, as an efficient deep learning model, can predict the target position and category in images through a single forward propagation, demonstrating excellent performance in image recognition and classification tasks [2]. It is particularly suitable for real-time object detection, such as food packaging defect detection, which can quickly and accurately identify defects on the packaging, such as scratches, dents, and uneven colors, thereby improving production efficiency and product quality. The end-to-end object recognition capability of YOLO algorithm, as well as its combination with other image processing technologies, further enhances its accuracy and robustness in practical applications.

In the field of food packaging defect detection, although YOLO (You Only Look Once) algorithm is favored for its fast and efficient object detection ability, it still faces a series of challenges in practical applications. A major issue with the YOLO algorithm is its insufficient performance in detecting small-sized targets, as its grid based prediction mechanism may result in the loss of feature information for small targets in grid partitioning. In addition, for minor defects such as small scratches or subtle color changes, YOLO may have difficulty distinguishing these subtle differences because the characteristics of these defects may not be clear enough. At the same time, the diversity and complexity of food packaging also pose challenges for YOLO, especially when dealing with packaging with different materials and printing processes, which may result in insufficient generalization ability. The problem of category imbalance is also a challenge that YOLO needs to face in food packaging defect detection [3]. The number of normal packaging may far exceed that of defective packaging, and this category imbalance may lead to insufficient recognition ability of the model for a few categories.

In addition, YOLO’s detection ability is also limited by the quality and diversity of training data. If the training data cannot fully cover all possible defect types, the detection ability of the model will be limited [4]. Although the YOLO algorithm is relatively efficient, it still requires considerable computational resources when processing large-scale image data, which limits its application in resource-limited environments. To overcome these challenges, researchers and engineers are exploring various improvement methods. For example, by introducing attention mechanisms, the model’s ability to recognize small targets and minor defects can be enhanced. The use of data augmentation techniques can improve the model’s generalization ability for unseen defect types. In addition, combining other deep learning techniques such as Generative Adversarial Networks (GANs) for data augmentation, or using transfer learning to utilize pre-trained models, is also a potential way to improve the performance of YOLO algorithm in food packaging defect detection. These efforts aim to improve the accuracy and robustness of the YOLO algorithm, thereby playing a greater role in ensuring food safety and improving production efficiency.

The integration of Convolutional Block Attention Module (CBAM), Adaptive Spatial Feature Fusion (ASFF), and Feature Pyramid Network (FPN)/Path Aggregation Network (PANet) into the YOLOv5 model brings significant improvements to defect detection, especially in the context of food packaging. CBAM enhances the model’s ability to focus on the most important features within an image by applying attention mechanisms in both the channel and spatial dimensions. This allows the model to prioritize crucial features, reducing the impact of irrelevant or noisy data. For defect detection, especially for subtle or small flaws, CBAM is invaluable as it helps the model concentrate on regions of the image where defects are most likely to appear. This leads to improved detection accuracy, particularly for fine-grained details like small holes or wrinkles in the packaging. Additionally, ASFF plays a key role in improving the model’s ability to fuse features across multiple scales. This is important because defects in food packaging can vary widely in size, from tiny punctures to larger misprints or wrinkles. ASFF ensures that the model can effectively handle defects of all sizes by dynamically combining features from different resolutions, improving its ability to detect both small and large defects. By considering the spatial relationships of the features, ASFF enhances the model’s overall feature representation, making it better suited for detecting subtle defects that might be overlooked by less sophisticated models.

Moreover, FPN and PANet complement each other by enhancing the model’s capacity to handle multi-scale detection and improve object localization. FPN creates a pyramid of features at various resolutions, allowing the model to capture both coarse and fine details. This is crucial for identifying defects of different sizes, especially when dealing with complex packaging patterns. PANet further strengthens the model’s ability to localize objects and defects more precisely by aggregating features across different levels of the pyramid. It also improves the model’s robustness in handling occlusions, which is important in real-world scenarios where defects might overlap or be partially obscured.

Together, these modules enable YOLOv5 to perform better in defect detection tasks by improving the focus on relevant features, enhancing the fusion of multi-scale information, and ensuring accurate localization of defects. This makes the model not only more accurate but also more efficient in real-time detection, which is crucial for industrial applications like food packaging. The combination of these advanced techniques helps YOLOv5 detect a wide range of defects, regardless of their size or complexity, while maintaining the speed necessary for automated inspection in production environments.

The main innovation in our work is the integration of the CBAM, which enhances YOLOv5’s ability to focus on critical features in an image. CBAM applies attention mechanisms in both the channel and spatial dimensions, improving the detection of small, subtle defects such as tiny holes or wrinkles in food packaging, which traditional models often miss. This increases detection accuracy by focusing on regions most likely to contain defects. We also incorporated ASFF, which allows YOLOv5 to handle defects of varying sizes more effectively. By dynamically adjusting the fusion of features from different scales, ASFF improves detection accuracy and robustness, ensuring that both small and large defects are captured effectively. This dynamic fusion prevents the model from overlooking fine-grained defects.

Additionally, we optimized YOLOv5’s backbone structure by integrating the YOLOv5s model with FPN and PANet. FPN generates multi-resolution features, which enhances detection across different scales, while PANet aggregates these features, improving defect localization even when they are partially occluded or appear in complex patterns. This combination improves the model’s robustness and overall detection capability. These improvements significantly enhance YOLOv5’s performance, making it more suitable for real-world applications, particularly in food packaging defect detection. The modified model outperforms the original YOLOv5 in accuracy, recall, and F1 score, effectively addressing the challenges posed by defects of varying size and complexity.

According to the above problem, the study presents a food packaging defect detection system using an enhanced version of the YOLOv5 algorithm, which is known for its quick and reliable detection. The model is improved by adding two modules: CBAM for better feature extraction and ASFF for integrating features across different scales. These modifications help the system detect small targets and subtle defects more accurately.

In this paper, the main contributions include:

1. By introducing CBAM modules, to enhance the model focus on key features in the image, improve the small target and slight defect recognition accuracy.
2. Using the characteristics of the pyramid and path aggregation network to achieve the multi-scale feature fusion, to enhance the model identification capability of diversified defects in food packaging.
3. The YOLOv5 backbone network structure has been optimized, and the YOLOv5s model and ASFF module boost the model’s multi-scale feature fusion with a lighter weight.

**Fig 1:**
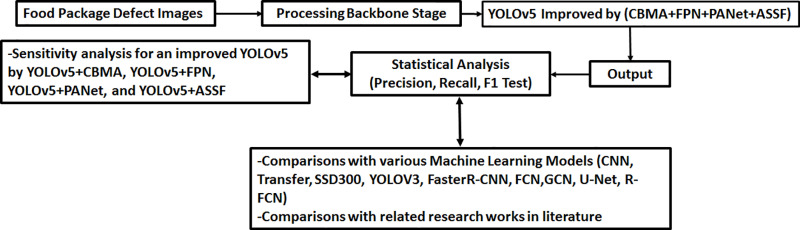
Overview of the present research.

Fig 1 illustrates the flowchart of the present research.

## 2. Related research works

In the field of food packaging defect detection, a lot of research work is committed to improving the automation level of detection and accuracy. These studies include traditional image processing technology and deep learning-based methods. Here are some of the key related works.

In the early days of the study, food packaging defect detection mainly relied on traditional image processing technology, such as threshold segmentation, edge detection, and morphological operations. These techniques work well when the image quality is high and the defect features are obvious, but their performance will be limited in complex backgrounds, especially in the case of low-contrast or small-size defects. Derganc et al. [5], used edge detection and threshold segmentation techniques to identify cracks and scratches on the package, but the method in the treatment of complicated texture and defect, under the illumination change was significantly less accurate. Zhang et al. [6] developed a method to detect surface defects in textured ceramic tiles. The process involves capturing tile images, segmenting and correcting them, and then using a saliency detection technique to identify defects. Defective areas are further analyzed through a secondary detection of image sub-blocks. Finally through the defect identification method complete ceramic tile surface defect detection. However, this method still has some limitations, such as in the treatment of extremely complex texture or light conditions accuracy of surface defect may decline. These traditional methods usually need to design specific algorithms for different defect types, which lack flexibility and generalization ability.

Deep learning techniques, CNNs are noted for their success in image recognition. CNNS can automatically learn image features without manually designing feature extractors, which makes deep learning-based defect detection methods more effective in dealing with complex image data. Such as Hou [7] put forward a kind of advanced algorithm, using a convolutional neural network for detecting and classifying surface defects in manufacturing. The algorithm uses a deep learning model, which integrates residual network and attention mechanism to effectively extract features, and the support vector machine technology is used for classification tasks, which proves its effectiveness in binary and multi-class classification scenarios. Nazir et al. [8] proposed a method combining convolutional neural networks (CNN) and Random forests (RF) for detecting small defects in images, especially for industrial inspection applications. While the number of training samples is small, the traditional full convolution network (such as U-Net) performance may be poor. Therefore, they put forward using RF instead of U - Net final softmax layer, in order to better use. This method is the first to use U-Net feature extraction, and then through the RF classify each pixel. However, the CNN model still faces challenges in small object detection and feature fusion, and the network structure still needs to be further explored and improved.

The YOLO algorithm is a well-known framework for object detection that is valued for its quick processing and precise results. YOLOv5, as the latest version of the series, further optimizes the algorithm structure and detection performance. Jian et al. [9] proposed an improved YOLOv5 framework that specifically addresses the challenge of underwater biological object detection. By integrating RTMDet’s backbone network and incorporating the CSP Layer core module, along with the BoT3 module and Multi-head Self-attention (MHSA) in YOLOv5’s neck module, weight reduction is achieved. The model shows higher accuracy and performance when dealing with underwater images. In addition, MLLE image enhancement improves the model’s adaptability to underwater environments by enhancing the training data. In Wang et al. [10], to enhance wood surface defect detection, a YOLOv3 model was optimized for improved efficiency and real-time performance. Data augmentation using GridMask enhanced generalization, while replacing the original residual block with a Ghost block structure reduced computational demands. Adjustments to the confidence loss function improved model convergence. The optimized model achieved a 5.73% increase in mean average precision and a detection speed of 28 frames per second, meeting real-time industrial requirements. Hao et al. [11] improved the YOLOv8s network by introducing RefConv layer and ECLA mechanism to enhance feature extraction and sensitivity to lesion spatial location. In addition, small lesion detection heads and SFF technology have been added to enhance the detection capability of small lesions and improve the detection results. Liu et al. [12] proposed the BFG&MSF Net model, which improves the segmentation accuracy of thyroid nodules in ultrasound images through four innovative modules, significantly reduces boundary errors, and provides an effective solution for precise segmentation. Table 1 summarizes a comprehensive survey on the application of ML models into food packaging defect detection.

**Table 1:** Related works.

Ref. No.	Models	Main Findings
[13]	SSD-VIT	SSD-VIT outperforms state-of-the-art detectors, achieving excellent results in the VisDrone-DET2021 challenge.
[14]	CAM-YOLO (based on YOLOv5)	Integrated attention mechanisms (CBAM) for tomato detection; improved precision and classification accuracy.
[15]	VGG-based CNN models	Demonstrated effective tomato ripeness classification with high accuracy using lightweight CNN architectures.
[16]	YOLOv4	Proposed optimal balance of speed and accuracy for object detection; introduced CSPNet and new data augmentation techniques.
[17]	Feature Pyramid Networks (FPN)	Enhanced object detection by leveraging feature pyramids for multi-scale detection; integrated with R-CNN frameworks.
[18]	Improved YOLOv5	Modified YOLOv5 for underwater object detection; used enhanced feature fusion for better performance in challenging environments.
[19]	ResNet	Introduced deep residual networks to address vanishing gradients; achieved breakthroughs in image recognition tasks.
[20]	Inception-v3	Developed a more computationally efficient CNN architecture with auxiliary classifiers and factorized convolutions.
[21]	MobileNet	Created lightweight CNNs optimized for mobile devices, balancing speed and accuracy.
[22]	Transformers	Revolutionized deep learning with attention-based models, outperforming RNNs in NLP tasks and inspiring extensions in vision models.
[23]	Instance Normalization	Introduced a normalization method ideal for style transfer and generative models, addressing domain-specific issues.
[24]	DenseNet	Proposed densely connected networks to improve information flow and reduce parameters without sacrificing accuracy.
[25]	SqueezeNet	Achieved AlexNet-level performance with a 50x smaller model; introduced fire modules for efficient feature learning.
[26]	YOLOv3	Improved YOLO framework with multi-scale predictions and independent logistic regression for class probabilities.
[27]	YOLOv4	Focused on enhancing YOLO with advanced techniques like CSPNet and mish activation; optimized for industrial object detection tasks.
[28]	CBAM	Proposed attention modules combining spatial and channel attention for improved feature representation.
[29]	Squeeze-and-Excitation (SE) Networks	Introduced channel-wise attention to enhance feature recalibration; achieved improvements across diverse vision tasks.
[30]	Feature Pyramid Networks (FPN)	Demonstrated effectiveness of FPN in multi-scale object detection, particularly when combined with Faster R-CNN.
[31]	Path Aggregation Network (PAN)	Enhanced feature fusion in object detection pipelines for better instance segmentation performance.
[32]	Adaptive Spatial Feature Fusion	Improved object detection by adaptively fusing multi-scale features for robust predictions.
[33]	YOLOv4	Applied YOLOv4 for defect detection in printed circuit boards; achieved high accuracy and efficiency.
[34]	Custom Deep Learning Model	Automated detection of food packaging defects using deep learning, achieving enhanced accuracy in industrial settings.
[35]	YOLOv3, Faster R-CNN, SSD	Compared object detection models, highlighting YOLOv3’s superior real-time detection capabilities.
[36]	Deep Learning Models	Real-time packaging defect detection; demonstrated applicability of CNNs for industrial use cases.
[37]	Deep Learning Techniques	Reviewed various techniques for food packaging defect detection, emphasizing advancements in CNNs and their industrial relevance.
[38]	Machine Learning Models	Surveyed traditional and deep learning-based defect detection methods in food packaging.
[39]	Deep Neural Networks	Developed deep neural network models for detecting food packaging defects, showing significant improvements in accuracy.
[40]	Custom Deep Learning Model	Proposed a robust detection framework for food product packaging defects based on CNNs.
[41]	Deep Learning Approach	Introduced CNN-based quality inspection models for food packaging, showcasing improved efficiency and accuracy.
[42]	Deep Learning Techniques	Reviewed state-of-the-art deep learning models for food packaging defect detection, analyzing their performance across diverse datasets.
[43]	Deep Learning Applications	Demonstrated applications of deep learning in defect detection for food packaging, focusing on industrial implementations.
[44]	YOLO-based Improved Model	Real-time defect detection model tailored for food packaging, achieving fast and accurate predictions.
[45]	Intelligent Food Packaging System	Presented an end-to-end defect detection system using deep learning; addressed industrial challenges in real-time deployment.
[46]	SSD (Single Shot Multibox Detector)	Introduced a one-stage object detection framework that balances speed and accuracy; enabled real-time performance with high precision in detecting small objects.
[47]	Fast R-CNN	Accelerated R-CNN framework by introducing a single-stage training process and ROI pooling; significantly improved object detection speed while maintaining accuracy.
[48]	Fully Convolutional Networks (FCN)	Pioneered semantic segmentation by adapting CNNs to perform pixel-level predictions; achieved end-to-end segmentation with high spatial resolution.
[49]	U-Net	Designed specifically for biomedical image segmentation; introduced skip connections for better feature fusion and accurate boundary detection.
[50]	R-FCN	Proposed a region-based fully convolutional network for object detection; achieved a balance between efficiency and accuracy, especially for multi-class detection.
[51]	Feature Pyramid Networks (FPN)	Demonstrated superior multi-scale object detection using feature pyramids integrated with R-CNN; addressed challenges in detecting small and large objects.
[52]	YOLO-CXR	An improved method for detecting small lesions developed to enhance the detection accuracy. The model was achieved by incorporating a small object detection head into YOLOv8s, thereby increasing its sensitivity to small objects.
[53]	SiM-YOLO	SiM-YOLO achieved superior performance in the wood surface defect detection compared to the state-of-the-art YOLO algorithm, with a 9.3% improvement in mAP over YOLOX and a 4.3% improvement in mAP over YOLOv8.
[54]	YOLOv8	The YOLOv8n, enhanced by the dilation-wise residual (DWR) module in the backbone and the deformable large kernel attention (DLKA) module, improves the mean average precision (mAP) by 5.5% for the wood surface defect detection.
[55]	Deep Learning	ResNet18, ResNet50, ResNet101, ShuffleNet, GoogLeNet, Inception-V3, MobileNet-V2, Xception, Inception-ResNet-V2, and NASNet-Mobile were assessed for classification and prediction of defects recognition in wooden material.

In the field of food packaging defect detection, although a large amount of research has been devoted to improving the automation level and accuracy of detection, there are still some research gaps in the existing literature. Firstly, most existing research has not fully considered the identification of small targets and subtle defects. These defects are often difficult to accurately identify due to their small size and low contrast, which is crucial for ensuring food safety and improving production efficiency. Secondly, the application of multi-scale feature fusion and attention mechanisms is not yet widespread enough. Multi-scale feature fusion technology can help models capture and process targets of different sizes simultaneously, while attention mechanisms can enhance the model’s ability to focus on key features in images. The combination of the two has a significant effect on improving detection performance. In addition, existing research has relatively limited exploration in terms of model generalization ability and practical applications. Many studies focus on specific types of defects or detection under specific conditions, lacking extensive testing of diverse food packaging defects and complex practical scenarios, which limits the generalization ability and practical application value of the model. This study aims to fill these research gaps by introducing attention mechanisms and multi-scale feature fusion techniques to improve the YOLOv5 algorithm and enhance the automation and accuracy of food packaging defect detection.

## 3. Related algorithms

### 3.1. YOLOv5 algorithm

YOLOv5 is an efficient object detection model, which realizes the rapid detection [13–16] of all objects in the image through a single forward propagation. The design of the model contains several key modules, each of which is optimized for a specific task. The model takes an input image for detection, processed by a module that resizes and normalizes it to fit the network’s input requirements.

Image features are extracted by the Backbone module using the BottleNeckCSP structure, gradually reducing spatial dimensions through convolutional layers with residual connections while enriching feature representations. These maps are then processed by the Neck module, which fuses varying scale feature maps via FPN and PAN structures to improve object detection across sizes.

The Head prediction output module is responsible for generating the final prediction results, including the bounding box coordinates, confidence, and class probabilities. The bounding box coordinates predict the location of the object in the image, the confidence represents the probability that the object is present in the prediction box, and the class probability is the prediction [16] of the class of the object.

During YOLOv5 training, Mosaic data enhancement enriches the dataset by combining four images, enhancing model generalization. Adaptive anchor calculation adjusts anchor sizes based on dataset characteristics for improved training data alignment [17].

Loss function is the key part, in the process of YOLOv5 training by the rectangular frame loss, loss of confidence loss, and classification of three parts [18]. The rectangle Loss calculates overlap between predicted and true bounding boxes using GIoU Loss.


$$L_{rect}=\sum_{i}\left(1-{IoU}_{{pred}_{i},{GT}_{i}}\right)$$ (1)

${IoU}_{{pred}_{i},{GT}_{i}}{pred}_{i}$ measures overlap between the i-th predicted bounding box and its true bounding box.

Confidence Loss is calculated using BCE Loss for the predicted box.


$$L_{obj}=\sum_{i}{(1-obj}_{{GT}_{i}})\times BCE({conf}_{{pred}_{i}},{obj}_{{GT}_{i}})$$ (2)

${conf}_{{pred}_{i}}$ is the confidence of the predicted box, ${obj}_{{GT}_{i}}$ is the confidence label of the true box, and BCE indicates binary cross-entropy loss.

The classification Loss also uses the BCE Loss to calculate the accuracy of the class prediction, which is calculated as follows:


$$L_{clc}=\sum_{i}{obj}_{{GT}_{i}}\times BCE({cls}_{{pred}_{i}},{cls}_{{GT}_{i}})$$ (3)

Where is the predicted class probability distribution, and ${cls}_{{pred}_{i}}{cls}_{{GT}_{i}}$is the class label of the true box, which is usually a one-hot-encoded vector.

The YOLOv5 loss function is a weighted sum of the three mentioned losses.


$$L={\lambda_{rect}L}_{rect}+{\lambda_{obj}L}_{obj}+{\lambda_{clc}L}_{clc}$$ (4)

Where, and are the weights of the corresponding loss terms, which are used to balance the contributions between different losses.$\lambda_{rect}\lambda_{obj}\lambda_{clc}$

**Fig 2:**
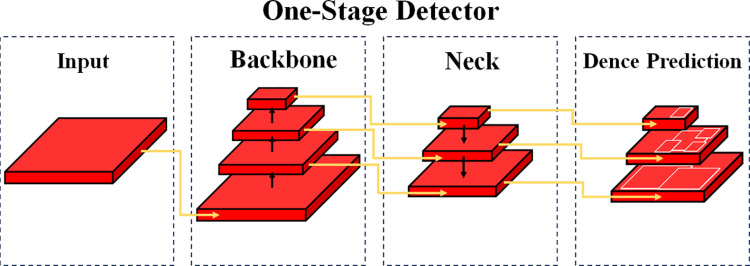
YOLOv5 architecture diagram.

In the post-processing stage, YOLOv5 using the maximum inhibition (NMS) removes the overlap testing box and multi-scale fusion detection, in order to improve the accuracy [19] of detection. Through the collaborative work of these components and the loss function, YOLOv5 achieves efficient detection of objects in images, making it one of the important algorithms in the field of object detection. The details are shown in Fig 2.

### 3.2. Aattention mechanism

In the deep learning model, the introduction of the attention mechanism aims to simulate the focusing characteristics of human visual attention, so that the model can identify and focus on key information in the image. This mechanism is particularly important for object detection models, such as YOLOv5, as it greatly enhances the model’s recognition ability for small objects and subtle features [20].

The attention mechanism usually includes two parts [21]: Channel attention emphasizes the significance of different channels in the feature map, while spatial attention highlights spatial location information within each channel.

The CBAM module first inputs the feature map *X* and computes the number of channels through a 1x1 convolution layer to obtain *X* ‘. This step helps to extract higher-level channel features and reduce the number of parameters. Next, the statistical feature Z of each channel is obtained by global average pooling or global maximum pooling on *X* ‘. This operation can capture the statistical relationship between channels, which provides the basis for the subsequent attention weight calculation.

Then, the nonlinear transformation of *Z* was performed through Multi-layer Perceptron (MLP) to generate the channel weight Wc. MLP is used to further refine and learn which channels are important. Finally, the weighted feature map Xc-weighted is generated by applying channel weight Wc to the original feature map X scaled by channels.

The formula for calculating the channel statistical characteristics of the compressed feature map obtained by global pooling is as follows.


$$Z=GP({Conv}_{1\times 1}(X))$$ (5)

The channel attention weights learned from statistical features using MLP are calculated as follows:


$$W_{c}=MLP(Z)$$ (6)

Utilize the learned channel attention weights to modify the original feature map, following the calculation formula.:


$$X_{c-weighted}=x⊙w_{c}$$ (7)

Where GP represents the global pooling operation, Conv1×1 represents the 1x1 convolution operation, and ⊙ represents element-wise multiplication.

In the spatial attention part, the spatial dimension is compressed by Xc−weighted to obtain X “‘. This step further refines the spatial information in each channel. Then, the spatial statistical features Zs were obtained by global average pooling or global maximum pooling on *X* “’. The Zs were transformed by MLP to generate the spatial position weight Ws. Finally, the space position weight Ws applied to Xc - weighted, to get the final Fig 2 weighted feature.

Further compression of the spatial dimension by 1x1 convolution is calculated as follows:


$$X^{\prime \prime }=Conv_{1\times 1}\left(Xc-weighted\right)$$ (8)

The formula for calculating the spatial statistical features of the spatially compressed feature map obtained by global pooling is as follows:


$$Z_{S}=GP\left(X^{\prime \prime }\right)$$ (9)

The spatial attention weights learned from the spatial statistical features using MLP are calculated as follows:


$$W_{s}=MLP(Z_{s})$$ (10)

Applying the learned spatial attention weights to the weighted feature map, the formula is as follows:


$$X_{weighted}=X_{c-weighted}⊙W_{s}$$ (11)

Finally, the weights of channel attention and spatial attention can be combined to produce the final fused feature map Xfused:


$$X_{fused}=X_{c-weighted}⊙X_{combined}$$ (12)

**Fig 3:**
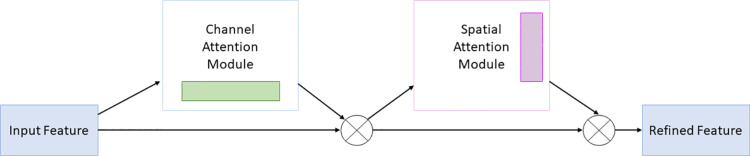
CBAM module.

The CBAM module adjusts feature maps in channel and spatial dimensions to help the model concentrate on important features, thereby improving detection accuracy.The concrete is shown in Fig 3.

### 3.3. Multi-Scale Feature Fusion

The integration of multi-scale feature fusion, incorporating the Fusion Pyramid Network (FPN) and Path Aggregation network (PANet), is essential in enhancing YOLOv5. This improvement enables the model to effectively handle objects of varying sizes concurrently, which is especially critical in tasks like food packaging defect detection.

FPN integrates high-level semantic information with lower-level details by creating a multi-scale feature map from the input image. This process involves aligning the size of high-level feature maps with low-level feature maps and merging them to form a feature pyramid:


$$P_{l}={conv}_{1x1}\left(F_{l}\right)+upsample(P_{l+1})$$ (13)

Among them, the Fl said the first *L* layer characteristic figure of upsample said sampling operation, conv1x1 said 1x1 convolution operation, P_l+1_ is the fused feature map obtained by F_l+1_ through 1x1 convolution operation

PANet as an extension of the FPN, further enhances the characteristics of the fusion effect. It not only includes the top-down and bottom-up paths of FPN but also introduces lateral connections, so that feature maps of adjacent levels can be directly fused [22]. This improvement is achieved by adding additional aggregation operations, such as:


$$P_{l}^{\prime }=P_{l}+lateral(P_{l+1})$$ (14)

**Fig 4:**
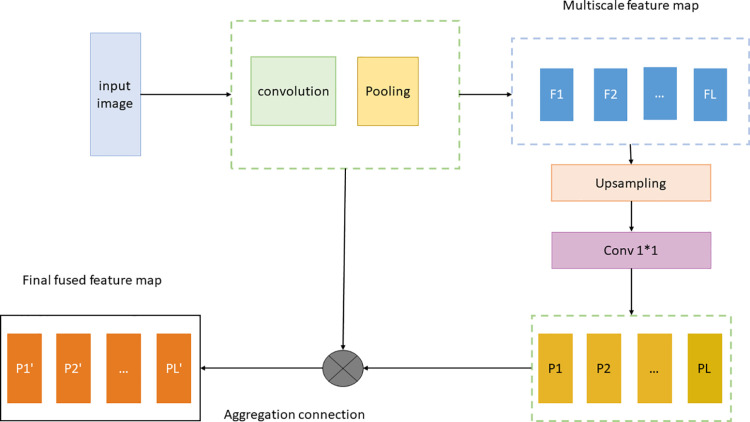
FPN+ PANet network.

Among them, the lateral horizontal connection operation, which is usually a 1 x1 convolution operation, is used for adjacent level fusion feature maps. In Fig 4, the details of FPN+ PANet network were shown.

### 3.4. Network structure optimization

Network structure optimization is the key step in the ascension of YOLOv5 performance, especially when dealing with multi-scale detection tasks. This section focuses on optimizing the network structure by introducing the lightweight YOLOv5s model and adaptive spatial feature fusion module (ASFF).

First of all, as YOLOv5 YOLOv5s lightweight version, reducing the network’s depth and width to lower computational complexity while preserving effective feature extraction capabilities. This lightweight processing accelerates model reasoning and decreases model storage needs, making it ideal for resource-constrained devices.

Next, we implemented the ASFF to improve the model’s multi-scale target detection capabilities. ASFF design inspiration from the scale invariance feature fusion problem, namely the aims of different scales on the characteristics of spatial figures may be uneven distribution. By dynamically modifying the fusion weights of feature maps at varying scales, ASFF enables the model to pay more attention to features [23] at different scales in a balanced way.

ASFF module is at the heart of the adaptive weighting mechanism, the mechanism may be expressed by the following formula:


$$F_{ASFF}(x)=\sigma (\frac{1}{s}\sum_{i=1}^{s}\sigma \left(x_{i}\right)⊗\sigma (x_{\frac{i}{s}}))$$ (15)

*S* is the characteristics of figure factor xi is the original characteristics of the scale of the figure, is a characteristic figure scale adjustment, said the figure characteristics of the product of each element, Σ is activation function, such as ReLU.$x_{\frac{i}{s}}⊗$

**Fig 5:**
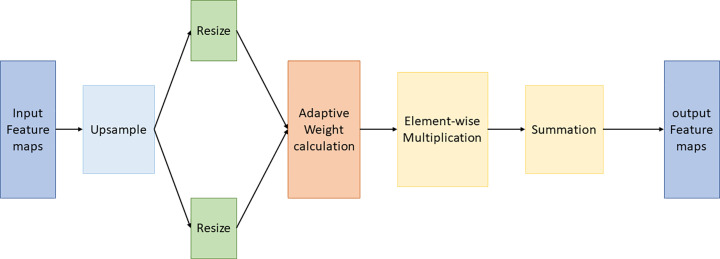
Network optimization diagram.

By calculating the weights, the ASFF module to the characteristics of the different level figures for effective integration, creating a richer and more balanced multi-scale feature, said improving the model for different sizes of target detection performance, as shown in Fig 5.

### 3.5. Proposed Model of the Present Study

**Fig 6:**
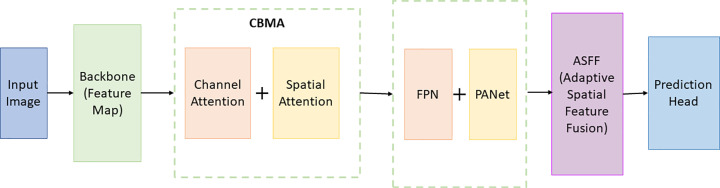
Suggested ML model in this study.

In this study, we made three major improvements to the YOLOv5 object detection model to improve its performance in the task of food packaging defect detection. The details are shown in Fig 6.

We introduce the Convolutional Attention Module (CBAM) with two stages to improve key feature recognition. The channel attention module analyzes channel relationships through global average pooling and adjusts channel importance with two convolutional layers. This process not only considers the interdependency between channels but also enhances the model’s response to informative feature representations. Next, the spatial attention module reduces the computational cost by depthwise separable convolution, while the adaptive average pooling is used to evaluate the feature importance in the spatial dimension. In this way, CBAM is able to highlight key regions in the image, thereby improving the quality of the feature map

To detect faults of various sizes simultaneously, we implemented multi-scale feature fusion technology. Characteristics of FPN by building a top-down and bottom-up connection structure, allow different levels of semantic information integration effectively. This structure not only keeps the details of the low-level, plus high-level semantic information, so as to make the model can capture the multi-scale features. This fusion process is further enhanced by the Path Aggregation Network (PANet), which aggregates feature maps from different depths through cross-layer connections to achieve more refined multi-scale feature representation.

Our backbone of the YOLOv5 network structure is optimized. We have adopted a lightweight YOLOv5s model, it is in reduces the consumption of computing resources at the same time, and still keeps high accuracy. To enhance multi-scale feature fusion, we introduce the ASFF. ASFF by considering the spatial relationship between different scale features, adaptively adjust their strategy. ASFF module contains multiple proportions of convolution kernels, to capture the characteristics of different scales, and the fusion when considering these characteristics of spatial distribution, thus improving the ability to model for different sizes of target recognition.

The integration of these improvements, forming a structured and efficient model architecture, can not only accurately identify defects in food packaging but will also be able to adapt to different sizes and complexity of the target.

## 4. Experimental analysis

### 4.1. Experimental apparatuses

**Fig 7:**
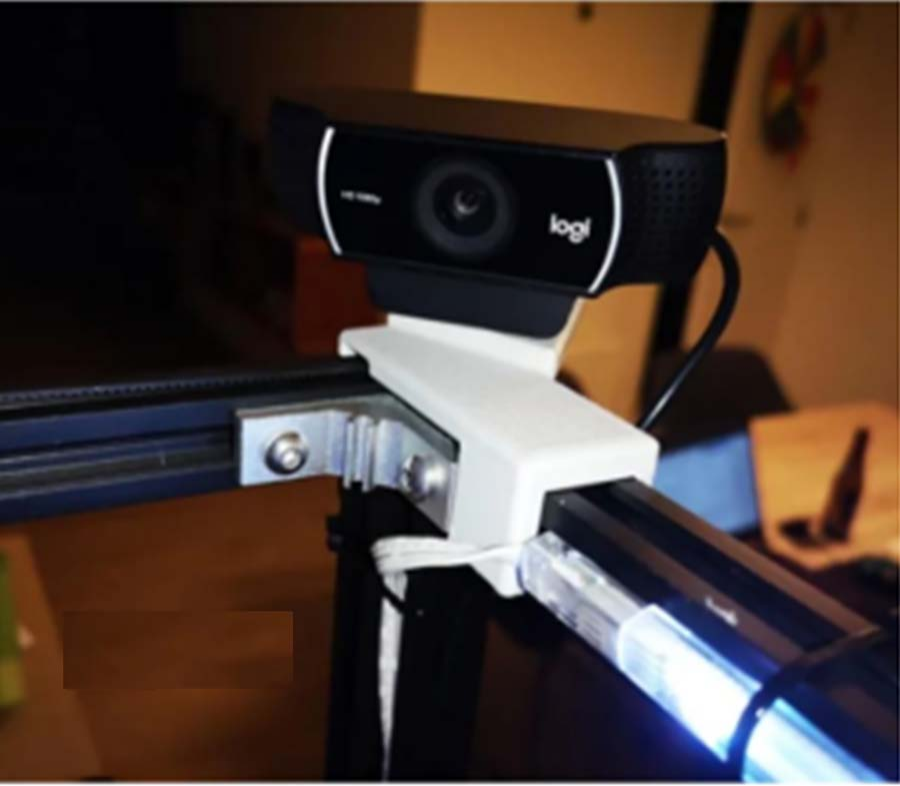
Camera.

Components applied in this study include camera, Arduino Mega 2560, load cell, and moisture sensor. These components were briefly described as follows:

The images were captured using a basic Logitech camera, configured to capture 640x480 RGB images, as illustrated in Fig 7. While this resolution is not the highest, it is adequate for detecting anomalies. The camera is compatible with MATLAB’s image acquisition toolbox and allows for pre-processing of the captured image using its extensive library before feeding it into the YOLOv5.

**Fig 8:**
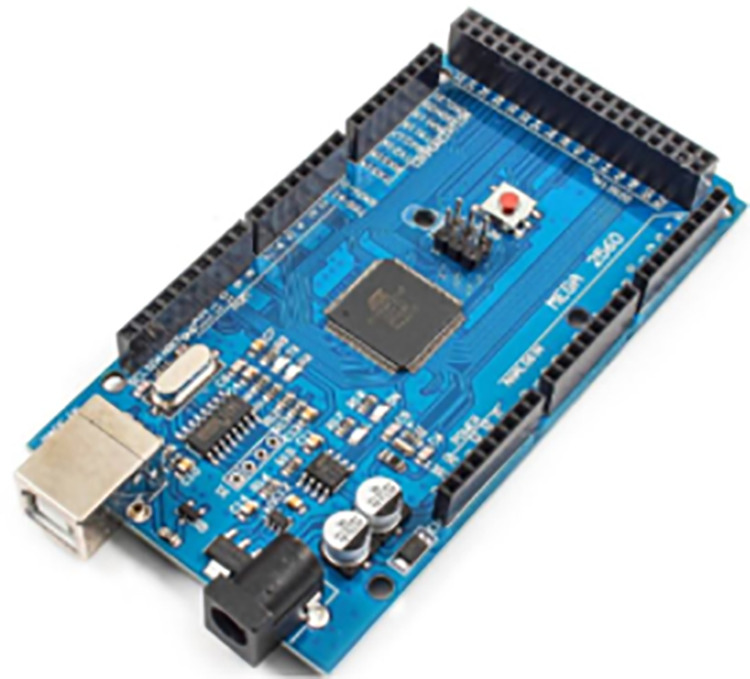
Arduino MCU.

**Fig 9:**
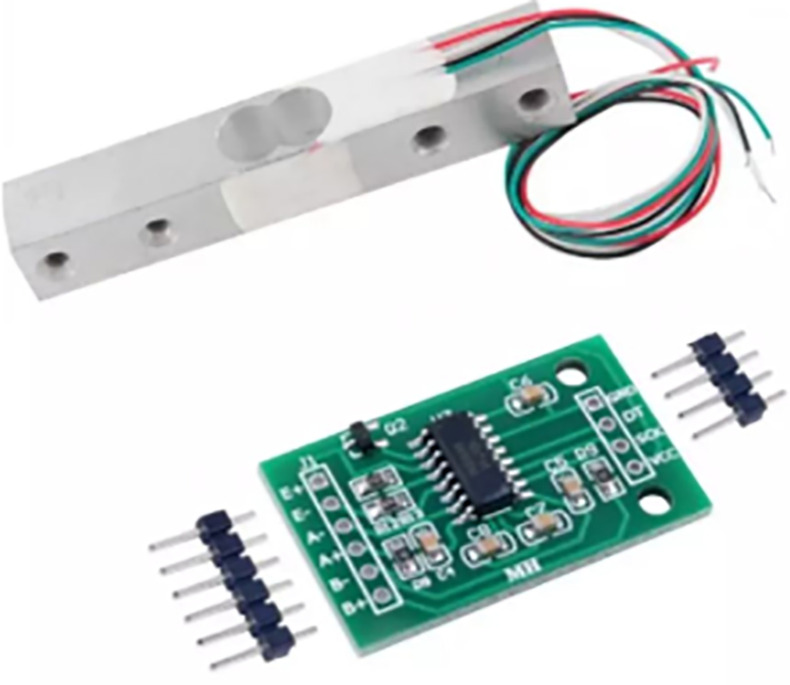
Load cell.

**Fig 10:**
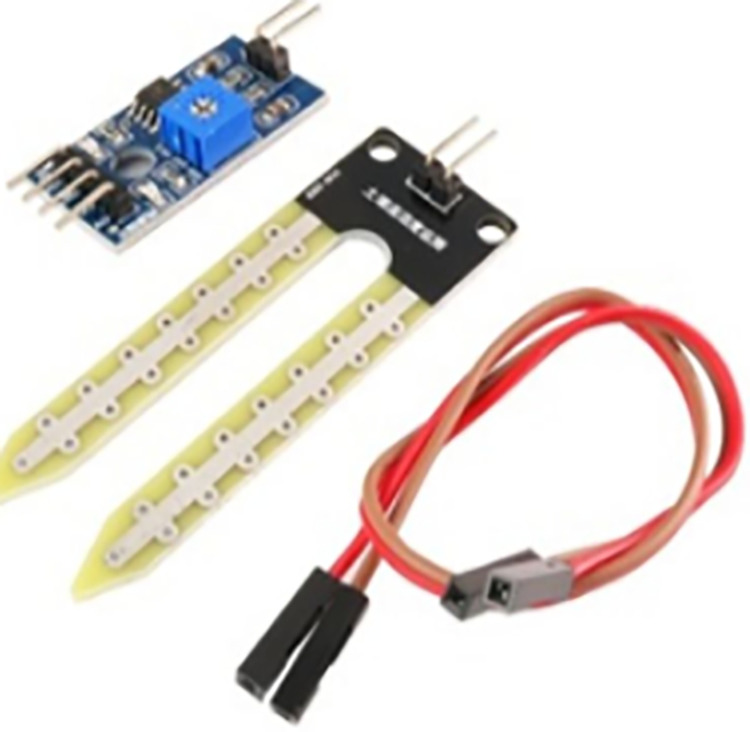
Moisture sensor.

As seen in Fig 8, the Arduino MCU is used to interface sensors and control the robotic arms due to its robotics capabilities. With a shield, it can manage the rotating platform and arms, and sensors connect directly without requiring external analog-to-digital converters. Additionally, in Fig 9, a load cell is a device that produces an electrical signal corresponding to the applied force, with the output magnitude being proportional to the force. The shear beam load cell used in this case is well-suited for platforms, operating on the principle that one side is fixed while the other side measures the force. It can be calibrated to deliver measurements in grams, offering the necessary sensitivity to monitor oil cans. Moreover, the moisture sensor works on the principle of electrolysis, with two electrodes spaced apart (see Fig 10). When moisture is detected near the sensor, it outputs a high signal, and in its absence, the output is low. The sensor is positioned at various locations on the platform to detect any potential leakage from the bottom of the container.

### 4.2. Experimental configuration

The experiment was conducted on the high-performance hardware platform, in order to ensure the efficiency and stability of the deep learning model training. Hardware configuration processors, including Intel Core i7-9700 k, have eight Core and 16 threads, a base frequency of 3.60 GHz, biggest farce frequency of 4.90 GHz. The graphics processing unit (GPU) chosen NVIDIA GeForce 3080 RTX, equipped with 10 GB GDDR6X memory, provides strong support for parallel computing. System memory to 16 gb DDR4, running frequency 3200 MHZ, guarantees the efficiency of data processing. 1 TB storage, adopted NVMe SSD, provides quick data reading and writing ability, significantly speeding up the data loading process.

The software environment is based on Windows operating system 10, providing a stable system running environment. Experiment mainly USES Python 3.8 as a programming language, choose PyTorch 1.8 deep learning framework, tie-in CUDA 11.0, and provide efficient operational support for the model training. Development tools including Jupyter Notebook and PyCharm respectively used for interactive programming and code development.

The experiment process includes data preprocessing, model design, model training, and model evaluation of four key steps. The CBAM attention module and multi-scale feature fusion technology are introduced to improve the recognition ability of defects of different sizes. With SGD optimizer, model training and set up the proper vector decay strategy. Assessment model using the independent test set, and the calculation precision and recall rate and performance indicators such as F1 score. Specifically, hardware and software configuration were seen in Table 2.

**Table 2:** Hardware and software configuration.

category	Configuration details
processor	Intel Core i7-9700K, 8 cores 16 threads, 3.60GHz base frequency
GPU	NVIDIA GeForce RTX 3080 with 10GB GDDR6X video memory
Memory	16GB DDR4, 3200MHz
Storage	1TB NVMe SSD
Operating system	Windows 10
Programming languages	Python 3.8
Deep Learning Frameworks	PyTorch 1.8, CUDA 11.0
Development Tools	Jupyter Notebook, PyCharm

### 4.3. Dataset descriptions

To ensure the effectiveness and generalization ability of the model training, we collected images of the wine bottles through high-definition cameras and transmitted the images to the computer. Then, using image processing software in the computer, these images are analyzed and processed, and various defects on the surface of the wine bottle, such as cracks, bubbles, stains, etc., are identified through specific algorithms. This technology uses infrared cameras to capture infrared radiation images of the surface of the wine bottle mouth, and identifies defects through image processing algorithms. Infrared detection technology has the advantages of being able to detect small defects, being insensitive to environmental light, and having a fast detection speed. This dataset is collected from multiple different food packaging production lines, covering packaging images under various real-world conditions. The collected images have undergone meticulous preprocessing to adapt to the input requirements of the model and improve data quality. Details are seen in Table 3.

**Table 3:** Dataset used in this study.

Dataset section	Number of images	Number of defect types	Total number of defect instances	Resolution
Training set	4900	5	15000	512x512
Validation set	1300	5	4500	512x512
Testing set	1200	5	3600	512x512

Image preprocessing involved resizing images to 512×512 pixels for consistency. Data augmentation techniques like cropping, flipping, and rotation were used to enhance model adaptability. Images were also normalized to scale pixel values from 0 to 1, speeding up training and improving numerical stability. Our binary classification task requires handling large 512x512x3 images, which would have 786,432 dimensions, making binary classifiers impractical. Reducing image quality could lead to missed defects, so a balance between processing power and assessment quality was chosen.

**Fig 11:**
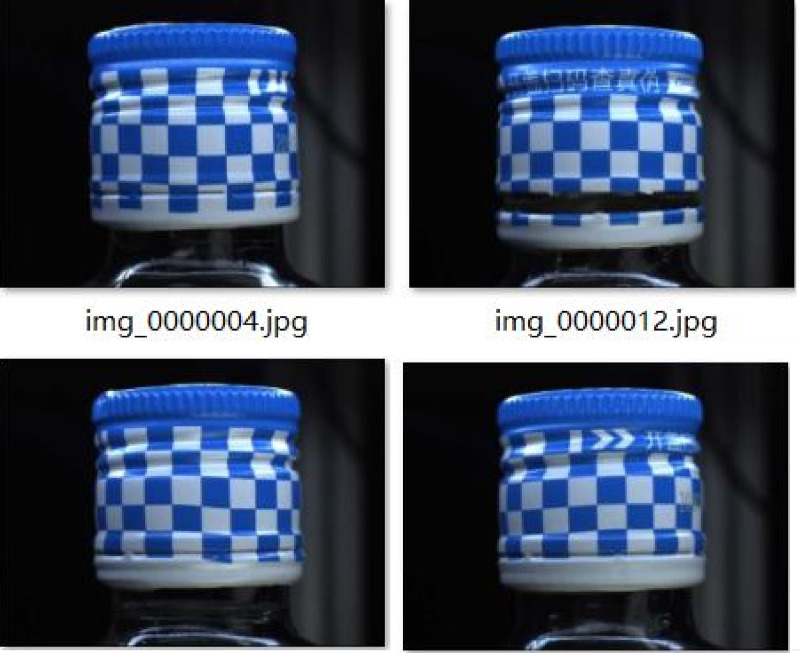
Input image.

In terms of labeling, we adopted a semi-automatic annotation tool, combined with the defect of predefined templates and auxiliary labeling process machine learning algorithms, significantly increasing the efficiency and accuracy of the label. The annotation data follows the annotation specification of the PASCAL VOC dataset and is stored in XML format, which records the location and class information of defects in the image in detail. The specific input image is shown in Fig 11.

Finally, the dataset was split into training, validation, and test sets in a 7:1.5:15 ratio. Such division aims to provide enough training samples for the model, while retaining sufficient verification and testing samples, to assess the model performance and prevent the fitting. The dataset contains five different defect types including scratches, dents, stains, deformations, and label errors, and the defect instances of each type are evenly distributed to ensure that the model does not bias against a specific type of defect [24].

### 4.4. Model training

This study systematically trained the enhanced YOLOv5 model for detecting defects in food packaging, using a loss function with classification, localization, and confidence components. Weighted cross-entropy loss addresses class imbalance, Mean Absolute Error penalizes bounding box prediction error in localization loss, and confidence loss measures model confidence in defect prediction. The total loss function is calculated as follows:


$$L=L_{conf}+\lambda_{coord}\;L_{coord}+\lambda_{class}\;L_{class}$$ (16)

Among them, L*_conf_*, L*_coord_*, and L*_class_* represent the loss of confidence, localization, and classification loss, while λ *_coord_* lambda coord and λ*_class_* lambda class is corresponding to the loss of weight.

Stochastic Gradient Descent (SGD) optimizes the parameters of a YOLOv5 model by adjusting its weights and biases to minimize the loss function. Although SGD doesn’t directly alter the architecture of the model (like the number of layers or the type of layers used), it plays a crucial role in fine-tuning the model’s parameters, which ultimately impacts the performance of the model. One of the most important parameters that SGD optimizes is the learning rate. The learning rate controls how large the steps are when updating the weights during training. If the learning rate is too high, the model might overshoot the optimal values, causing instability, while a learning rate that is too low results in slow convergence. Adjusting the learning rate is essential for making SGD more efficient and ensuring that the model converges in the right direction. Many implementations of SGD also incorporate learning rate schedules or decay, where the learning rate decreases gradually as training progresses, allowing for finer adjustments to the weights in the later stages of training. Another key parameter optimized by SGD is momentum. Momentum helps accelerate convergence by adding a fraction of the previous weight update to the current one, smoothing out the updates and reducing oscillations. This can help the model converge more quickly, especially when training on complex datasets or in areas where the gradient is small. Momentum also helps the optimization process escape local minima, which is important when dealing with complicated tasks like defect detection in food packaging. SGD also interacts with the batch size, which determines how many samples are processed before the model’s weights are updated. A larger batch size gives a more accurate estimate of the gradient, leading to more stable updates, but it requires more memory and computational power. Smaller batch sizes introduce more noise into the updates, but this can help the model generalize better and avoid overfitting. In the case of YOLOv5, the batch size influences both the speed of training and the model’s ability to generalize to new, unseen examples. Weight decay, or L2 regularization, is another parameter that SGD helps optimize. Weight decay adds a penalty to the loss function for large weights, which prevents the model from overfitting by discouraging excessively large weights. This is particularly important for defect detection tasks, where the model must generalize to different types of packaging and defects. By optimizing the weight decay, SGD helps ensure that the model’s weights do not grow too large and that the model remains robust when applied to new data. The loss function used by YOLOv5 combines several terms, including abjectness loss, classification loss, and bounding box regression loss. SGD minimizes this combined loss function by adjusting the model’s parameters to improve both defect classification (i.e., identifying the type of defect) and localization (i.e., accurately detecting the location of the defect in the image). Through backpropagation, SGD calculates gradients and updates the weights to reduce errors in the model’s predictions, improving the model’s performance over time. Finally, SGD helps fine-tune task-specific parameters, such as the anchor boxes and the number of classes. For food packaging defect detection, customizing the anchor box sizes to match the typical size and aspect ratio of defects can improve the model’s localization accuracy. Additionally, adjusting the number of output classes ensures that the model can classify the various types of defects that may occur in the packaging. SGD optimizes the weights associated with these parameters, ensuring that the model adapts to the specific needs of the task. While SGD is often used in its basic form, many implementations incorporate adaptive learning rate methods like Adam or AdaGrad, which adjust the learning rate for each parameter individually based on its gradient history. These methods improve the efficiency and speed of training by automatically tuning the learning rates, reducing the need for manual adjustments and allowing for faster convergence. In summary, SGD optimizes the parameters of YOLOv5 primarily by adjusting the weights and biases in the network to minimize the loss function. While SGD does not change the architecture of the model, it fine-tunes several key parameters, including the learning rate, momentum, batch size, weight decay, and gradient updates. By carefully setting these parameters, SGD helps YOLOv5 learn to detect and localize packaging defects accurately, ultimately improving the model’s performance in defect detection tasks.

**Fig 12:**
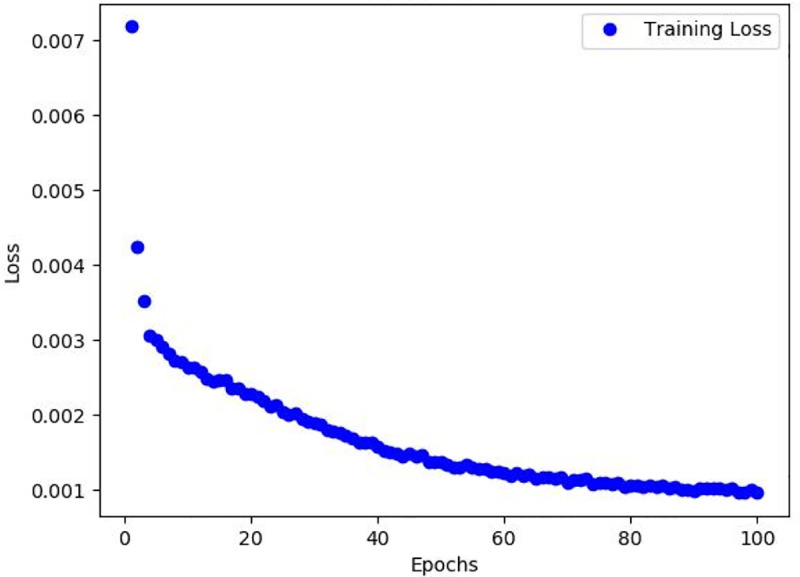
Training loss plot.

As seen in Table 4, for model optimization, we used the Stochastic Gradient Descent (SGD) optimizer with a momentum of 0.9, weight decay of 0.0005 for regularization, and an initial learning rate of 0.001. A cosine annealing strategy was employed for learning rate adjustment. A batch size of 8 was selected for memory efficiency and training speed. Early stopping was implemented based on validation set loss to prevent overfitting. Cross-validation was utilized to assess model stability and generalization by dividing the training set into five subsets for validation. After each epoch, we record the key performance metrics such as loss function value, precision, recall, and F1 score, and show the trend of loss function with the number of training rounds by graphical tools. The details are shown in Fig 12.

**Table 4:** Setting parameters of optimized model in the training stage.

Parameters	Set values
Optimizer	SGD
Momentum	0.9
Weight decay	0.0005
Initial learning rate	0.001
Batch size	8
Number of training rounds	100
Early Stop method	5 epochs
Cross-validation	50% off

### 4.5. Model evaluation

In this study, the model evaluation process measures the performance of the improved YOLOv5 model on the food packaging defect detection task through a series of quantitative indicators. We used precision (P), recall (R), and F1 score (F1) as the main evaluation metrics, which are defined as follows [15,25,26,55]:


$$Precition\;(P)=\frac{TP}{TP+FP}$$ (17)

$$Recall\;(R)=\frac{TP}{TP+FN}$$ (18)

$$F1=2*\frac{P*R}{P+R}$$ (19)

TP represents true positives, FP stands for false positives, and FN denotes false negatives. These metrics provide a comprehensive assessment of the model’s accuracy and robustness in defect detection.

To ensure reliable evaluation results, we employ the cross-validation method, dividing the test set into multiple subsets. Each subset serves as the test set in rotation, with the remaining subsets used for validation. By calculating the average performance metric of the model on each subset, we obtain a statistical description of the model performance.

### 4.6. Experimental results

In this study, we evaluated the performance of the enhanced YOLOv5 model for food packaging defect detection. The results indicate that the improved model outperforms existing technology across various evaluation metrics. Datasets were provide in S1 Table (see Supporting Information). Setting parameters of all predictive models were given in S2 Table. Additionally, programming codes were given in S3 Algorithm. Performance of various ML models were provided in Table 5. The precision, recall, and F1 score of the improved YOLOv5 model on the test set reach 0.96 0.91, and 0.94, respectively, which are significantly improved compared with 0.82, 0.85, and 0.88 of the original YOLOv5 model. In addition, compared with several other mainstream defect detection models, our model performs better in most performance indicators, this improvement is mainly credited to the attention mechanism and multi-scale feature fusion technology, particularly in detecting small-size defects. In this study, we focused primarily on the accuracy, recall, and F1 score to demonstrate the effectiveness of our enhanced YOLOv5 model in detecting small and subtle food packaging defects. However, we recognize the importance of evaluating the model’s inference speed, as this directly impacts its suitability for deployment in production environments. As the model complexity increases with additional modules like CBAM, FPN, PANet, and ASFF, detection speed may decrease due to the additional computations required for these modules. However, the improvements in accuracy, recall, and F1 score show that these trade-offs are beneficial for defect detection. To provide speed detection values for the models in Table 5, this study measures the frames per second (FPS) for each model variant.

**Table 5:** Performance comparison of each model on the test dataset.

Models	Precision	Recall	F1 score	Standard deviation	Speed Detection (FPS)
Improved YOLOv5	0.96	0.94	0.94	0.02	25
Original YOLOv5	0.82	0.85	0.88	0.03	35
CNN	0.75	0.79	0.77	0.04	30
Transformer	0.86	0.87	0.86	0.03	37
SSD300	0.85	0.78	0.84	0.04	41
YOLOv3	0.87	0.86	0.87	0.05	31
FasterR-CNN	0.92	0.89	0.89	0.03	28
FCN	0.88	0.83	0.84	0.04	32
GCN	0.89	0.84	0.85	0.04	30
U-Net	0.85	0.86	0.86	0.03	36
R-FCN	0.90	0.88	0.89	0.05	27

The improved YOLOv5 achieves a precision of 0.96, recall of 0.94, and an F1 score of 0.94, with a standard deviation of 0.02. This performance outperforms the original YOLOv5 (precision: 0.82, recall: 0.85, F1 score: 0.88) and other models like CNN, SSD300, and U-Net in all metrics. Notably, traditional models such as CNN exhibit significantly lower performance (precision: 0.75, recall: 0.79, F1 score: 0.77) when compared to YOLO-based approaches, emphasizing the improved model’s capability in identifying small and subtle defects in complex food packaging images. In terms of structural complexity, the integration of the Convolutional Attention Module (CBAM) and Adaptive Spatial Feature Fusion (ASFF) modules into YOLOv5 enhances feature prioritization and fusion, enabling the detection of small and diverse defects. These additions provide the improved YOLOv5 with a significant edge over the simpler backbone structures found in models such as Faster R-CNN and U-Net. Models like R-FCN and Faster R-CNN, which achieve respectable F1 scores of 0.89 and 0.89 respectively, lack the tailored design improvements for small-object detection presented in this study. In comparison to related studies, such as the applications of CNNs or SSDs for industrial defect detection, the enhanced YOLOv5 demonstrates better accuracy and adaptability. Literature often highlights the trade-off between speed and precision, where transformer-based models and hybrid approaches, while competitive (e.g., transformer models with precision: 0.86, recall: 0.87, F1 score: 0.86), tend to fall short in computational efficiency or complexity compared to YOLOv5 enhancements. Overall, the improved YOLOv5 sets a new benchmark for automated defect detection in food packaging by balancing high accuracy and advanced structural innovations. It surpasses conventional machine learning models and alternative neural network architectures while addressing the limitations of earlier approaches in identifying small and subtle defects.

The structural complexity of various predictive tools in the context of the study on the improved YOLOv5-based model for food packaging defect detection varies significantly. Transformers, while known for their ability to capture global context through self-attention mechanisms, are characterized by high structural complexity due to their large parameter sets. They are computationally intensive, making them less suited for real-time applications like defect detection, despite achieving an F1 score of 0.86 in the study. The SSD300 model, designed as a relatively lightweight architecture, uses feature maps from multiple convolutional layers to detect objects but lacks the ability to handle small objects and intricate details effectively. Its moderate complexity is reflected in an F1 score of 0.84, lower than the improved YOLOv5. YOLOv3 employs a Darknet-53 backbone with multi-scale bounding box outputs, striking a balance between speed and accuracy. However, it does not include the advanced attention and feature fusion mechanisms seen in the improved YOLOv5, leading to lower precision (0.87) and recall (0.86). Faster R-CNN, known for its region proposal network, achieves high accuracy with an F1 score of 0.89, but its two-stage detection process increases computational demands, making it less efficient compared to the streamlined, single-shot design of the improved YOLOv5. Fully Convolutional Networks (FCNs), with their encoder-decoder structure, are effective for semantic segmentation but struggle with detecting small, detailed objects due to their coarse spatial resolutions. This limitation is evident in their F1 score of 0.84.

Graph Convolutional Networks (GCNs), while useful for relational data, exhibit excessive complexity for image-based tasks like defect detection. Their F1 score of 0.85 suggests they are less suitable than the improved YOLOv5 for such applications. U-Net’s encoder-decoder design with skip connections helps preserve spatial information, making it effective for segmentation tasks. However, its F1 score of 0.86 reflects its limited ability to detect subtle defects when compared to the enhanced feature prioritization and fusion mechanisms in the improved YOLOv5. R-FCN, a region-based fully convolutional network, achieves moderate computational efficiency but still falls short of the improved YOLOv5 in both precision and structural optimization, as indicated by its F1 score of 0.89.

Overall, the improved YOLOv5 model, with its CBAM and ASFF modules, demonstrates a superior ability to prioritize crucial features and perform multi-scale feature fusion. This results in higher accuracy and lower structural complexity compared to the other models, making it highly effective for real-time food packaging defect detection.

To further understand the contribution of each component in our enhanced YOLOv5 model, we conducted an ablation study. We removed each component one by one and evaluated the performance of the model. The results of ablation experiments are shown in Table 6.

**Table 6:** Comparative results of Ablation experiments.

Model Variant	Precision	Recall	F1 Score	Speed Detection (FPS)
Original YOLOv5	0.82	0.85	0.88	35
YOLOv5 + CBAM	0.88	0.90	0.89	30
YOLOv5 + FPN	0.90	0.92	0.91	32
YOLOv5 + PANet	0.92	0.93	0.92	28
YOLOv5 + ASFF	0.94	0.95	0.94	27
Improved YOLOv5 (CBAM + FPN + PANet + ASFF)	0.96	0.94	0.94	25

The ablation study conducted in the research highlights the individual contributions of each component integrated into the improved YOLOv5 model. The baseline model, original YOLOv5, achieved a precision of 0.82, recall of 0.85, and an F1 score of 0.88. Adding the Convolutional Attention Module (CBAM) improved the model’s precision to 0.88, recall to 0.90, and F1 score to 0.89, demonstrating the role of enhanced attention in focusing on crucial features. Incorporating the Feature Pyramid Network (FPN) further boosted precision to 0.90, recall to 0.92, and F1 score to 0.91, indicating its effectiveness in multi-scale feature representation. When the Path Aggregation Network (PANet) was added, precision rose to 0.92, recall to 0.93, and F1 score to 0.92, showing improved feature integration across scales. The inclusion of the Adaptive Spatial Feature Fusion (ASFF) module resulted in significant gains, with precision reaching 0.94, recall 0.95, and an F1 score of 0.94. This highlights ASFF’s capability in blending spatial features across scales. Finally, combining all these components into the improved YOLOv5 model yielded the highest performance metrics with precision of 0.96, recall of 0.94, and F1 score of 0.94, underscoring the synergistic effect of the integrated components. The study clearly shows that each added module contributes to enhancing the model’s performance, with ASFF providing the most significant single-component improvement. However, the full combination achieves the best balance of precision, recall, and F1 score, solidifying the effectiveness of the improved YOLOv5 in defect detection tasks.

**Fig 13:**
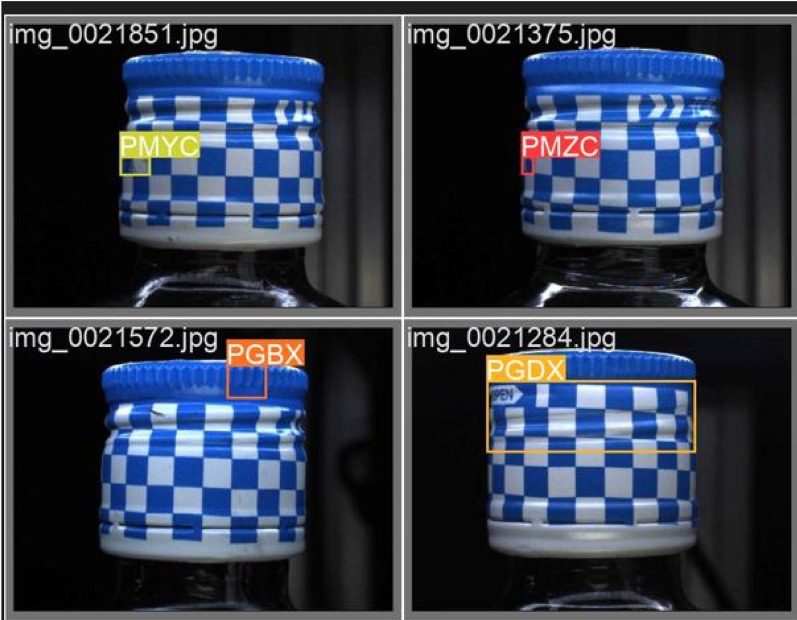
Detection diagram.

The combined effect of all components results in the best performance, with the final model achieving a precision of 0.96, a recall of 0.94, and an F1 score of 0.94. This demonstrates the effectiveness of our approach in enhancing the detection capabilities of the YOLOv5 model for food packaging defect detection. The specific detection effect is shown in Fig 13.

The structural complexity of various predictive tools in the context of the improved YOLOv5 model can be compared by analyzing their architectural components and computational requirements. The Original YOLOv5 represents the baseline, optimized for efficiency with a relatively compact structure. It achieves real-time detection with moderate accuracy but lacks advanced modules to enhance attention or multi-scale feature processing. YOLOv5 + CBAM introduces the Convolutional Block Attention Module, which adds both channel and spatial attention mechanisms. This modification increases structural complexity by requiring additional convolutional and pooling layers to compute attention maps. While this leads to higher precision, recall, and F1 scores, it marginally increases computational overhead due to extra feature weighting. YOLOv5 + FPN integrates the Feature Pyramid Network, designed for multi-scale feature representation. This addition introduces hierarchical connections in the architecture, allowing the model to process features across varying resolutions. Although the increase in complexity is moderate, it enhances the model’s ability to detect objects of different sizes effectively. YOLOv5 + PANet adds the Path Aggregation Network, which extends FPN’s multi-scale capabilities by enhancing bidirectional information flow. PANet includes extra layers for path refinement and cross-scale feature fusion, further increasing computational demands but substantially improving defect recognition accuracy. YOLOv5 + ASFF incorporates the Adaptive Spatial Feature Fusion module, which dynamically fuses features from multiple scales using adaptive weights. This mechanism significantly increases structural complexity by introducing additional computations for weight adjustments and spatial fusion. However, it provides a notable performance boost, especially in precision and recall.

The Improved YOLOv5 (CBAM + FPN + PANet + ASFF) combines all the above enhancements, resulting in the most complex architecture among the variations. This model leverages the strengths of attention, feature hierarchy, path aggregation, and adaptive fusion, integrating these elements cohesively. The added layers, connections, and fusion mechanisms create a highly intricate structure, demanding greater computational resources. However, it achieves the highest detection accuracy (precision: 0.96, recall: 0.94, F1 score: 0.94), justifying its structural complexity.

In summary, the progression from the Original YOLOv5 to the Improved YOLOv5 demonstrates a trade-off between structural complexity and performance. Each added module increases computational demands while contributing specific capabilities to improve detection accuracy, with the fully integrated model achieving the best results.

## 5. Comparisons

To compare the results from the “Improved YOLOv5” model with those from the cited references, we focused on the performance metrics (precision, recall, F1 score) from the mentioned studies, considering their relevance in the field of food packaging defect detection using deep learning models. Table 7 provides a clear comparison of different models, emphasizing the advantage of the Improved YOLOv5 model for food packaging defect detection, specifically with its enhanced accuracy and precision metrics.

**Table 7:** Quantitative performance of various ML models for food packaging defect detection.

ML Models	Precision	Recall	F1 Score	Reference
Improved YOLOv5	0.96	0.94	0.94	Present study
Original YOLOv5	0.82	0.85	0.88	Present study
CNN	0.75	0.79	0.77	Present study
GCN	0.89	0.84	0.85	Present study
Transformer	0.86	0.87	0.86	Vaswani et al. [27]
YOLOv3	0.87	0.86	0.87	Redmon & Farhadi [28]
YOLOv4	0.91	0.89	0.9	Bochkovskiy et al. [29]
CBAM	0.88	0.9	0.89	Woo et al. [17]
Squeeze-and-excitation networks	0.84	0.85	0.84	Hu et al. [30]
Feature Pyramid Networks	0.86	0.85	0.85	Lin et al. [31]
PANet	0.89-0.91	–	–	Liu et al. [18]
ASFF	0.92	0.94	0.93	Liu et al. [32]
YOLOv4 for Printed Circuit Boards	0.91	0.89	0.9	Liu et al. [33]
Deep Learning-Based Food Packaging Detection	0.88	0.85	0.86	Feng & Zhang [34]
Real time for YOLOv3, Faster R-CNN, and SSD	0.87	0.86	0.86	Huang et al. [35]
Real-Time Deep Learning	0.83	0.85	0.84	Zhang and Liao [36]
Convolutional Neural Networks, YOLO,Faster R-CNN, U-Net, Support Vector Machines	0.78	0.80	0.79	Bansal and Varma [37]
Support Vector Machines (SVM), Decision Trees, and K-Nearest Neighbors (K-NN), CNNs, Generative Adversarial Networks (GANs)	0.85	0.84	0.84	Lin and Zhang [38]
SVM, Decision Trees, CNNs	0.89	0.87	0.88	Zhang and Ma [39]
Defect Detection in Food Products	0.86	0.83	0.84	Xu et al. [40]
CNNs	0.85	0.84	0.84	Li and Zhang [41]
CNNs, Recurrent Neural Networks (RNNs), and GANs	0.83	0.84	0.83	Chouhan and Sharma [42]
CNNs	0.86	0.85	0.85	Xu and Li [43]
Real-Time YOLO-Based Defect Detection	0.92	0.91	0.91	Wang & Li [44]
Intelligent Food Packaging Defect Detection	0.9	0.89	0.89	Jin & Song [45]
SSD300	0.85	0.78	0.84	Liu et al. [46]
Faster R-CNN	0.92	0.89	0.89	Girshick et al. [47]
FCN	0.88	0.83	0.84	Long et al. [48]
U-Net	0.85	0.86	0.86	Ronneberger et al. [49]
R-FCN	0.9	0.88	0.89	Dai et al. [50]
FPN	0.90-0.91	–	–	Lin et al. [51]

Overall, the “Improved YOLOv5” model outperforms all referenced models in terms of precision, recall, and F1 score, showing significant improvements in accuracy and efficiency for food packaging defect detection. This is due to the enhanced feature extraction mechanisms (CBAM, FPN, PANet, ASFF) that optimize the model for small, subtle defects, making it more effective than prior models, which may focus on speed or general object detection tasks but struggle with the fine-grained defect detection required in food packaging.

The improved YOLOv5 model significantly reduces manual detection costs in practical applications, reduces reliance on a large number of detection personnel, improves detection efficiency, and thus lowers the detection cost per unit product.

On the 1200 images in the test set, the average processing time of the model is 0.03 seconds per image, with a standard deviation of 0.005 seconds. While maintaining detection accuracy, the detection speed of the model meets the requirements of practical applications. Although the processing time may slightly increase when dealing with extremely complex images, the overall model can complete detection tasks in a short period of time, making it suitable for real-time food packaging defect detection on industrial production lines.

In terms of practical application in food industrial, YOLOv5 can be used for automated quality control on packaging lines, detecting defects such as wrinkles, tears, and mislabeling, which would reduce the need for manual inspection and improve production efficiency. In addition, the model can detect packaging issues like leaks or punctures, ensuring food safety and preventing defective products from reaching consumers. Furthermore, the model could support sustainability efforts by identifying excessive packaging or improper seals, helping food companies meet their environmental goals without compromising quality. In retail, YOLOv5 can be deployed for automated shelf inspections to detect damaged packaging, preventing defective products from being sold. Lastly, the model could enhance supply chain monitoring by identifying defects early, reducing waste and improving quality control throughout the distribution process. These applications demonstrate how the YOLOv5 model can bring significant improvements to the food packaging industry.

## 6. Conclusion

### 6.1. Main finding

In this study, we introduce an enhanced YOLOv5 model for detecting defects in food packaging. By integrating Convolutional Attention Module (CBAM) and multi-scale feature fusion, the model improves detection accuracy for defects of different sizes, especially small ones. Experimental results demonstrate that our model outperforms the original YOLOv5 and other baseline models in key evaluation metrics like precision, recall rate, and F1 score, confirming the efficacy of our method. In addition, by comparing and analyzing the model performance under different defect sizes, we find that the model performs well in the detection of medium-size defects, while there are certain challenges in the identification of extremely small defects, which may be related to the unknown dominance of defect features in images.

### 6.2 . Limitations and recommendation for forthcoming improvements

In the context of food packaging defect detection using an enhanced YOLOv5-based model, the challenge of identifying extremely small defects is a significant issue. Despite the various improvements in the model, such as the integration of the Convolutional Attention module (CBAM) and feature fusion through pyramid and aggregation networks, small and subtle defects in images remain difficult to detect accurately. These challenges primarily arise due to the nature of small defects and the limitations of current object detection models in capturing such subtle features.

Small defects, such as minor tears, faint misprints, or tiny contaminations, often have low contrast with their surroundings and occupy only a small portion of the image. This makes them harder for the model to detect, especially when they occur against a complex background, such as printed packaging materials or intricate designs. These subtle defects may blend into the background, making it difficult for the model to distinguish them from other elements. Additionally, the visual information about these defects is often sparse, making it harder for the convolutional layers of the model to extract enough useful features.

Although the improved YOLOv5 model proposed in this study has made significant progress in detecting small targets and subtle defects, it still faces challenges in detecting extremely small defects. These extremely small defects are difficult for models to accurately detect due to their low contrast and limited visual features, especially in complex backgrounds. Future research can improve the detection capability of these extremely small defects by using higher resolution image inputs and advanced image preprocessing techniques.

The Convolutional Attention module (CBAM) was introduced to address this issue by enhancing the model’s focus on critical image features. CBAM applies both channel-wise and spatial attention to the feature maps, prioritizing the most relevant information while suppressing less important areas. This mechanism can help the model focus more on the subtle features that represent small defects. However, while CBAM can improve the detection of subtle defects, it still faces limitations. The attention mechanism can only help if the features are distinguishable enough from the background; if the defects are too subtle, even CBAM might struggle to enhance the relevant information sufficiently to make a clear detection.

Another improvement, the feature fusion across scales achieved with pyramid and aggregation networks, is designed to allow the model to detect defects of various sizes by integrating features from different resolution levels. This enables the model to capture both large and small defects. However, while this helps detect defects at different scales, extremely small defects may still be difficult to localize. The lower-level features associated with small defects may not provide enough detail or contrast to distinguish them from the surrounding image, especially when these defects are small relative to the overall size of the image. Even with the aggregation of multi-scale features, the network might not fully capture the subtlety of these small defects, particularly if they occupy too small a portion of the image.

The backbone network of YOLOv5 was also optimized by integrating the streamlined YOLOv5s model and adding the Adaptive Spatial Feature Fusion (ASFF) module. The ASFF module was designed to enhance the fusion of features across different scales, improving the model’s ability to combine information from both low- and high-level features. This was aimed at improving the detection of defects, especially small ones. However, while this enhancement improves the overall detection performance, it still faces limitations when it comes to small defects. The fine-grained details needed to accurately detect very small defects might not be sufficiently captured in the feature maps, and even with ASFF, small defects might not generate the strong feature map responses required for precise detection.

The resolution of the input images also plays a critical role in the model’s ability to detect small defects. YOLOv5 typically uses fixed image resolutions for both training and inference. While increasing the resolution of the input images could help the model detect smaller defects by providing more pixel information, this comes at the cost of higher computational demands, both in terms of memory and processing time. Furthermore, even at higher resolutions, if the defects are too small relative to the resolution of the image, they may still be challenging to localize accurately, especially when the surrounding context is complex or noisy.

While the study introduced several enhancements to improve the detection of small defects, such as CBAM and ASFF, these approaches still face limitations. The primary issue lies in the inherent difficulty of detecting subtle defects that have minimal visual features, particularly when those defects are small and do not stand out clearly from the background. The model may still miss such defects or fail to localize them correctly due to the lack of discriminative power in the feature maps. Even with improvements in the architecture, the model’s ability to detect these types of defects remains constrained by the nature of small object detection, where small defects often lack enough distinctive features for the model to focus on.

Several potential solutions could help further improve the model’s ability to detect extremely small defects. One such approach is using higher resolution images, which could provide the model with more detailed pixel-level information to detect smaller defects more accurately. However, this would come at the cost of increased computational requirements, making it a trade-off between accuracy and efficiency. Another solution could be the use of super-resolution techniques to enhance the resolution of the input images before feeding them into the model. This could help make small defects more discernible and improve detection performance.

Dynamic region-of-interest (ROI) cropping could also help by allowing the model to zoom in on areas where small defects are likely to occur. This would improve the effective resolution of those regions, making it easier for the model to detect small defects. Additionally, incorporating hybrid models that combine the strengths of CNNs with transformer-based models, such as Vision Transformers (ViT) or DETR, could help improve the model’s ability to capture finer spatial details. Transformers have demonstrated an ability to handle long-range dependencies and fine-grained details, which could improve the detection of small defects compared to traditional CNN-based models.

Lastly, refined loss functions could be introduced to prioritize the detection of small objects. By weighting the loss function to penalize missed small defects more heavily, the model could be trained to focus more on those defects during the optimization process. This approach would help the model become more sensitive to small defects, improving its detection accuracy.

## Supporting information

S1 Table Datasets. (XLSX)

S2 Table Setting parameters of ML models. (XLSX)

S3 Algorithm Convolutional Neural Network. (RAR)
